# Examining the relationships between body image, eating attitudes, BMI, and physical activity in rural and urban South African young adult females using structural equation modeling

**DOI:** 10.1371/journal.pone.0187508

**Published:** 2017-11-16

**Authors:** Alessandra Prioreschi, Stephanie V. Wrottesley, Emmanuel Cohen, Ankita Reddy, Rihlat Said-Mohamed, Rhian Twine, Stephen M. Tollman, Kathleen Kahn, David B. Dunger, Shane A. Norris

**Affiliations:** 1 MRC/WITS Developmental Pathways for Health Research Unit, Department of Paediatrics, School of Clinical Medicine, Faculty of Health Sciences, University of Witwatersrand, Johannesburg, South Africa; 2 MRC/Wits Rural Public Health and Health Transitions Research Unit, School of Public Health, Faculty of Health Sciences, University of the Witwatersrand, Johannesburg, South Africa; 3 INDEPTH Network, Accra, Ghana; 4 Umeå Centre for Global Health Research, Umeå University, Umeå, Sweden; 5 Department of Paediatrics, MRL Wellcome Trust-MRC Institute of Metabolic Science, NIHR Cambridge Comprehensive Biomedical Research Centre, University of Cambridge, Cambridge, United Kingdom; University of Zurich, SWITZERLAND

## Abstract

The persistence of food insecurity, malnutrition, increasing adiposity, and decreasing physical activity, heightens the need to understand relationships between body image satisfaction, eating attitudes, BMI and physical activity levels in South Africa. Females aged 18–23 years were recruited from rural (n = 509) and urban (n = 510) settings. Body image satisfaction was measured using Stunkard’s silhouettes, and the 26-item Eating Attitudes questionnaire (EAT-26) was used to evaluate participants’ risk of disordered eating. Minutes per week of moderate to vigorous physical activity (MVPA) was assessed using the Global Physical Activity Questionnaire (GPAQ). Significant linear correlates were included in a series of regressions run separately for urban and rural participants. Structural equation modeling (SEM) was used to test the relationships between variables. Urban females were more likely to be overweight and obese than rural females (p = 0.02), and had a greater desire to be thinner (p = 0.02). In both groups, being overweight or obese was positively associated with a desire to be thinner (p<0.01), and negatively associated with a desire to be fatter (p<0.01). Having a disordered eating attitude was associated with body image dissatisfaction in the urban group (β = 1.27, p<0.01, CI: 0.38; 2.16), but only with a desire to be fatter in the rural group (β = 0.63, p = 0.04, CI: 0.03; 1.23). In the SEM model, body image dissatisfaction was associated with disordered eating (β = 0.63), as well as higher MVPA participation (p<0.01). These factors were directly associated with a decreased risk of disordered eating attitude, and with a decreased desire to be thinner. Findings indicate a shift in both settings towards more Westernised ideals. Physical activity may provide a means to promote a healthy body image, while reducing the risk of disordered eating. Given the high prevalence of overweight and obesity in both rural and urban women, this study provides insights for future interventions aimed at decreasing adiposity in a healthy way.

## Introduction

As modernisation increases globally, ideologies and perceptions about health and the body shift accordingly. Body image disorders are largely regarded as issues of the Western world, yet recent findings demonstrate their expansion beyond culturally-bound borders [1, 2]. Body image dissatisfaction and eating disorders are considered a global manifestation of a distressed state during adolescence [3]. This critical life period is potentially influenced by underlying depressive symptoms generated by social pressures, loneliness, feelings of helplessness and a lack of orientation exposing adolescents and young adults, particularly girls, to these mental disorders [3, 4]. In many low- to- middle- income countries (LMICs), stoutness has traditionally symbolised good health [5]; with overweight signifying beauty, happiness, health, and affluence in black South African women [6]. Additionally, in the African context where the prevalence of HIV/AIDS is high, being thin is frequently associated with the presence of disease [6–8]. However, with the high degree of westernisation and urbanisation occurring in LMICs, the prevalence of such disordered behaviours has been increasing in these settings [9–12].

Over the last two decades, some studies have been conducted in South Africa showing associations between eating disorders and body image satisfaction. In a comparative epidemiological study between Caucasian and non-Caucasian college students, Legrange et al [1] highlighted the emerging incidence of eating disorders among black South African girls, potentially caused by body weight concerns. Furthermore, urban-rural differences have been detected for eating behaviours in young adult black South African females—with urban females presenting with more disordered eating behaviours and weight management attempts. However, in this study, both urban and rural females were presenting with a high prevalence of body image dissatisfaction and eating attitudes indicating risk of an eating disorder [12]. More recent quantitative studies have also reported body image dissatisfaction and eating disorders among black adolescent women in South Africa. For example, an urban cross-ethnical study in adolescent girls [7] showed that black female adolescents had higher BMIs and increased risk of eating disorders compared to their white/mixed ancestry counterparts. However, in a recent study examining rural adolescent girls, those who desired to be fatter had significantly higher BMIs than those that desired to be thinner, and when presented with an underweight silhouette they perceived it as unhappy and weak [13].

Obesity and overweight are increasing rapidly in South Africa, while physical activity is decreasing with urbanisation [14]; however the effect of this transition on the development of body image dissatisfaction and eating disorders remains unclear. Furthermore, in light of the persistence of food insecurity coupled with over- and under-nutrition in South Africa [15], it is unclear how eating disorders may be influenced by the development of obesity and modern aesthetic criteria. Indeed, in African transitional countries, food insecurity could involve specific eating disorders such as binge eating disorders due to poor access to food resources [16, 17]. In conjunction, poor nutritional knowledge has been observed in both rural and urban young South African females [18]. Furthermore, one of the behaviours often associated with eating disorders and body image dissatisfaction is extreme exercising [19], and this weight management strategy has previously been observed in a South African population [12]. Therefore, although obesity and physical inactivity tend to go hand-in-hand as already evident in South Africa, normal-weight and underweight individuals experiencing body image dissatisfaction and disordered eating may abnormally increase physical activity levels in order to further control their body mass index (BMI), and thus their body image satisfaction [20, 21]. Alternatively, it is possible that if physical inactivity is persisting with body image dissatisfaction, moderate exercise could be encouraged as a means to improve body image satisfaction. The relationship between body image satisfaction, eating attitudes, BMI and physical activity is evidently varied and dependent on setting and culture, age and gender; as are the behaviours employed by individuals to address these perceptions and eating attitudes [22]. Hence in this study we aimed to: (1) identify the differences in body image satisfaction and eating attitudes between rural and urban South African young adult females and; (2) explore the interactions between BMI, physical activity, body image satisfaction and eating attitudes using site specific regressions and structural equation modeling.

## Materials and methods

### Study design and setting

This cross-sectional study was conducted at two sites in South Africa: in the rural Agincourt area of Bushbuckridge municipality, Mpumalanga Province, where the Medical Research Council/Wits University Rural Public Health and Health Transitions Research Unit runs the Agincourt health and socio-demographic surveillance system (Agincourt HDSS), and in urban Greater Soweto, Johannesburg, Gauteng. For the purpose of this study, and according to common global definitions, urban is defined as an area with a population >1000 and a population density >1000 per km^2^, while any area not considered urban is considered rural. The study sites have been described in detail elsewhere [23, 24], but briefly—the rural site consists of 31 individual villages (totaling over 110,000 inhabitants) spanning an area of 475 km2. There is poor road infrastructure, no piped water, rudimentary sanitation, and poor access to health facilities. The urban Soweto site (200 km2) is densely populated, and the majority of households have good access to piped water, sanitation facilities, and health care. In both sites, populations are mainly black South Africans. For this study, data were collected from December 2012 to July 2013 in Agincourt, and from March 2012 to December 2014 in Soweto.

### Participants

In the rural Agincourt site, females between the ages of 18–23 were selected from the existing 2011 Agincourt HDSS population. Of the 2126 potential females in the survey database, 996 were located during the data collection period and were invited to participate. Ultimately, 509 female participants provided consent and completed data collection for inclusion in the study. In the urban site, 510 young adult women were randomly selected from the Birth-to-Twenty plus (BT20+) cohort study (protocol described elsewhere—[24]), from the sample of 720 females who participated in the Young Adult Survey. All participants provided written consent to participate in the study, and were not mentally or physically disabled (assessed via observation by research nurses). The study protocols were approved by the Human Research Ethics Committee of the University of the Witwatersrand (Clearance certificates n°M120138 for the Ntshembo-Hope Cross Sectional Survey in Agincourt and n°M111182 for the BT20+ survey).

### Variables

All measurements performed in the rural and urban areas were standardised, and research teams were trained centrally to further ensure that data collection was harmonised across both sites.

#### Anthropometry

Trained research assistants according to standardised techniques performed all anthropometric measurements. Standing height was measured to the nearest millimetre using a calibrated Stadiometer (Holtain Stadiometer®, Crymych, UK). Weight was measured to the nearest 0.1kg with participants wearing light clothing using the Tanita Tanita model TBF-410 (Arlinghton Heights; USA) digital scale. BMI was calculated as weight (kg)/height^2^(m).

#### Socio-demographics

Participants’ were asked to self-report their age, highest level of attained education, and socio-economic status (SES) using an interview-administered questionnaire. An SES score was generated by summing the number of assets owned in the household from the following options: TV, car, washing machine, fridge, phone, radio, microwave, cell phone, DVD/Video, DSTV (cable channel), computer, internet access, and medical aid. A sum of assets score has been shown to be useful in determining socioeconomic status [25], and this particular score, based on the Demographic Health Survey for developing countries (see https://dhsprogram.com), has been successfully used in South Africa previously [26].

#### Body image satisfaction

Body image satisfaction was measured using Stunkard’s silhouettes, which have previously been used in South African adolescents in rural and urban environments [22]. Nine body silhouettes (coded from 1 to 9; with 1 being the thinnest and 9 being the fattest) were individually and randomly presented. Participants were required to select the silhouette which best represented their current body shape (feel figure), as well as that which best represented the body shape they desired for themselves (ideal figure). The Feel-Ideal Discrepancy score (FID) was then calculated by subtracting the ideal figure value from the feel figure value. A FID score of zero represented body satisfaction, a positive score represented a desire to be thinner, and a negative score represented a desire to be fatter [13]. Thereafter, all 9 silhouettes were placed in front of participants and they were asked to associate specific characteristics, such as “best”, “worst”, “clumsy”, “strongest”, etc. with a silhouette. The silhouettes were then grouped as described previously [19] into four categories for assessing associations with characteristics chosen: underweight (silhouettes 1 and 2), normal weight (silhouettes 3, 4, and 5), overweight (silhouettes 6 and 7), and obese (silhouettes 8 and 9).

#### Eating attitudes

The 26-item Eating Attitudes questionnaire (EAT-26) was used to evaluate participants’ risk of a future eating disorder. The EAT-26 has previously been used in both rural and urban South Africans [22, 27]. The EAT-26 consists of 26 questions, which are scored on a Likert scale from 0 (never, seldom, or sometimes) to 3 (always). The responses are then summed to obtain an overall score ranging from 0–78. A score greater than 20 represents a risk for developing a future eating disorder, and participants were thus categorised as being at risk or not [13].

#### Physical activity

Physical activity was assessed using the Global Physical Activity Questionnaire (GPAQ), and minutes per week of moderate to vigorous physical activity (MVPA) was calculated by adding occupational, travel-related and leisure time moderate and vigorous physical activity. Compliance with WHO physical activity guidelines [28] was assessed by classifying participants who met 150 minutes of moderate or vigorous activity per week and/or 75 minutes of vigorous activity per week as ‘Active’, and those who did not meet these guidelines as ‘Insufficiently active’. Furthermore, participation in leisure time physical activity was considered independently.

### Statistical analysis

All statistical analyses were done using STATA 13 for Mac (STATA Corp, USA). All young adults who reported that they were pregnant at the time of data collection were excluded from the analysis. Students’ unpaired t-test or chi-square tests were performed to compare characteristics between rural and urban participants. Bivariate linear regressions were performed for urban and rural participants separately to determine associations between FID score, EAT-26 score and potential confounders. Significant linear correlates (excluding any collinear variables) were then included in a series of multinomial logistics regressions and multivariate regressions run separately for urban and rural participants with FID score and EAT-26 score as the outcomes respectively. FID score was further compared between urban and rural participants who participated in leisure time physical activity.

Structural equation modeling (SEM) was used to test and estimate the relationships between physical activity, BMI, FID score and EAT-26 for the group as a whole, while controlling for SES and age to correct for differences between the groups. The structural equation model consists of two parts, the structural model and the measurement model. The structural model defines the direction of the relationship between composite latent variables (not applicable for this dataset) and other observed variables, while the measurement model presents the relationships between included variables. Direct, indirect and total effects were computed and recoded. To evaluate the best fitting model for our data, we calculated and recorded multiple goodness of fit indices including the Chi-squared test, standardised root mean squared residual (SRMR) and comparative fit index (CFI). Although Chi-squared test is commonly used to assess goodness of fit, it is highly sensitive to sample size [29, 30] and it is also often inflated with non-normal data. We therefore used the Hu and Bentler’s Two-Index Presentation Strategy (1999) combination rule with a cut off value of CFI of 0.90 or higher and a SRMR of 0.09 or lower considered as best fit [31]. Distribution of attributes associated with body image silhouettes were compared between groups using chi-square tests. Cronbach Alpha test was used to determine internal consistency of the EAT-26 questionnaire in the rural and urban sites separately. Values ≥0.7 were considered good and those between 0.6 and 0.7 were considered acceptable [32]. All data are presented as means and standard deviations (SD) or totals and percentages, and a p-value of <0.05 was considered significant in all cases.

## Results

Participant characteristics are presented in Table 1. Urban females had a higher SES score (p<0.01), and had 6% more overweight and 9% less normal weight participants than the rural females. Urban females had a higher average FID score, indicating a desire to be thinner, yet the distribution within FID categories was not different from the rural group (42% vs 47% desiring to be thinner, 31% vs 32% satisfied, and 27% vs 21% desiring to be fatter respectively, p = 0.06). Rural females had a higher average EAT-26 score, and also had higher numbers of females with a score >20, signifying a risk for eating disorder. The Cronbach Alpha for the EAT-26 questionnaire was 0.78 in the urban group and 0.64 in the rural group (items with particularly poor consistency were “I avoid (try not to eat) foods with sugar in them”, “I think about burring up calories/kilojoules (energy) when I exercise”, and “I engaged in dieting behaviour (try to lose weight)”. Total MVPA (mean(SD)) was higher in rural than urban participants (1800(56)min/week vs 707(38)min/week, p<0.001), as was total leisure time physical activity (p<0.001); however when excluding those who reported no leisure time physical activity (n = 382 in urban and n = 201 in rural participants) participation in leisure time physical activity became higher in urban than rural participants (293(23)min/week vs 219(17)min/week, p<0.001). There was no difference in FID score or EAT-26 score for those who reported leisure time physical activity compared to those who did not in either rural or urban participants.

**Table 1:** Characteristics of the urban and rural sample.

	Rural (n = 476)	Urban (n = 492)	
	Mean (SD) or %	Mean (SD) or %	p-value
**Age (years)**	21 (1.3)	23 (0.5)	<0.01
**Height (cm)**	161.5 (6.7)	159.9 (6.2)	<0.01
**Weight (kg)**	64.6 (14.0)	64.7 (15.6)	0.91
**SES (sum of household assets)**	5.6 (1.9)	8.8 (2.4)	<0.01
**BMI (kg/m**^**2**^**)**	24.8 (5.2)	25.3 (5.9)	0.14
*BMI Category*			0.02
*Underweight (%)*	5	7	
*Normal (%)*	56	47	
*Overweight (%)*	23	29	
*Obese (%)*	16	17	
**FID (score/2)**	0.37 (1.74)	0.64 (1.69)	0.02
**EAT-26 (score/78)**	13.68 (7.87)	10.70 (8.55)	<0.01
*Score>20 (%)*	23	12	<0.01
**Pregnancy before interview (%)**			<0.01
*Yes*	65	55	
*No*	35	45	
**Highest education level (%)**			<0.01
*Studied post high school*	20	50	
*Finished high school*	37	17	
*High school or primary school*	43	33	
**Sufficiently active (%)**			<0.01
*Yes*	97	77	
*No*	3	23	
**Marital status (%)**			<0.01
*Single*, *divorced or widowed*	26	54	
*In a relationship*, *cohabiting or married*	74	47	

Results of the regressions of predictor variables on FID score for rural and urban females presented in Table 2 showed that in the urban group, being overweight or obese was positively associated with a desire to be thinner, and negatively associated with a desire to be fatter; while being underweight was positively associated with a desire to be fatter. Having an EAT-26 score greater than 20 was positively associated with a desire to be thinner. Similarly, in the rural group, being overweight or obese was positively associated with a desire to be thinner and negatively associated with a desire to be fatter. Having an EAT-26 score greater than 20 was positively associated with a desire to be fatter. The results of the regressions of predictor variables on EAT-26 score presented in Table 3 show that in the urban group, both desire to be thinner and desire to be fatter were positively associated with a higher EAT-26 score. In the rural group, none of the predictor variables were associated with EAT-26 score.

**Table 2:** Multinomial regression showing the predictors of FID category in the urban and rural groups.

FID category	Urban Group			Rural Group		
	B coefficient	P value	95% CI	B coefficient	P value	95% CI
**Desire to be thinner**						
*Physically active*	0.08	0.76	-0.47; 0.65	0.29	0.68	-1.10; 1.69
*Insufficiently physically active (reference)*						
*BMI underweight*	-0.93	0.25	-2.51; 0.65	-14.68	0.98	-1380.32; 1350.97
*BMI normal (reference)*						
*BMI overweight or obese*	1.92	<0.01*	1.43; 2.40	1.78	<0.01*	1.28; 2.29
*Eat-26 score>20*	1.27	<0.01*	0.38; 2.16	0.03	0.92	-0.58; 0.64
**Desire to be fatter**						
*Physically active*	0.23	0.50	-0.44; 0.91	0.75	0.31	-0.70; 2.20
*Insufficiently physically active (reference)*						
*BMI underweight*	1.36	<0.01*	0.48; 2.24	0.45	0.34	-0.48; 1.39
*BMI normal (reference)*						
*BMI overweight or obese*	-1.92	<0.01*	-2.92; -0.93	-1.21	0.01*	-1.94; -0.48
*Eat-26 score>20*	1.04	0.06	-0.06; 2.14	0.63	0.04*	0.03; 1.23

* p<0.05

**Table 3:** Regression showing the predictors of EAT-26 score in the urban and rural groups.

Eat score	Urban Group			Rural Group		
	B coefficient	P value	95% CI	B coefficient	P value	95% CI
*Physically active*	0.10	0.92	-1.70; 1.89	3.67	0.11	-0.80; 8.14
*Insufficiently physically active (reference)*						
*BMI underweight*	1.92	0.22	-1.16; 5.00	0.84	0.64	-2.65; 4.34
*BMI normal (reference)*						
*BMI overweight or obese*	0.49	0.60	-1.36; 2.34	0.52	0.57	-1.26; 2.30
*Desire to be thinner*	4.47	<0.01*	2.57; 6.37	0.93	0.34	-0.96; 2.82
*Satisfied (reference)*						
*Desire to be fatter*	3.03	<0.01*	0.78; 5.29	1.29	0.19	-0.66; 3.23

* p<0.05

Table 4 shows the distribution of body silhouettes and their attributes as chosen by urban and rural females. All categories were significantly different between groups. While most participants overall thought the normal weight silhouette was best, more rural than urban females thought the underweight silhouette was best. In both sites the majority of females thought the normal weight silhouette portrayed the most respect; however, more females in the rural group thought the overweight and obese silhouettes portrayed the most respect and more urban females thought the obese silhouette portrayed the least respect. Rural and urban females thought the obese and normal weight silhouettes respectively appeared strongest. There was agreement between participants in both sites that the underweight silhouette appeared weakest. While most urban females thought the normal weight silhouette was happiest, there was more distribution amongst rural females between the underweight, normal weight and overweight silhouettes being happiest. The majority of urban and rural females associated the normal silhouettes with positive attributes; however negative attributes were mostly associated with obese silhouettes by the urban females and with obese and underweight silhouettes equally by the rural females.

**Table 4:** Percentage distribution of body sillhouette attributes for urban and rural females.

Silhouettes	Urban	Rural	P value
Best			<0.01
*Underweight*	8	16	
*Normal*	86	74	
*Overweight*	6	6	
*Obese*	0	4	
Worst			<0.01
*Underweight*	26	23	
*Normal*	0	5	
*Overweight*	1	2	
*Obese*	73	70	
Clumsy			<0.01
*Underweight*	24	18	
*Normal*	3	7	
*Overweight*	6	10	
*Obese*	67	65	
Most respect			<0.01
*Underweight*	12	12	
*Normal*	72	49	
*Overweight*	11	23	
*Obese*	5	16	
Least respect			<0.01
*Underweight*	41	58	
*Normal*	3	11	
*Overweight*	6	11	
*Obese*	50	20	
Strongest			<0.01
*Underweight*	4	9	
*Normal*	48	27	
*Overweight*	24	23	
*Obese*	24	41	
Weakest			<0.01
*Underweight*	87	79	
*Normal*	1	5	
*Overweight*	2	5	
*Obese*	10	11	
Happiest			<0.01
*Underweight*	8	13	
*Normal*	74	64	
*Overweight*	9	16	
*Obese*	9	7	
Unhappy			<0.01
*Underweight*	27	35	
*Normal*	2	10	
*Overweight*	4	8	
*Obese*	67	47	

**Fig 1:**
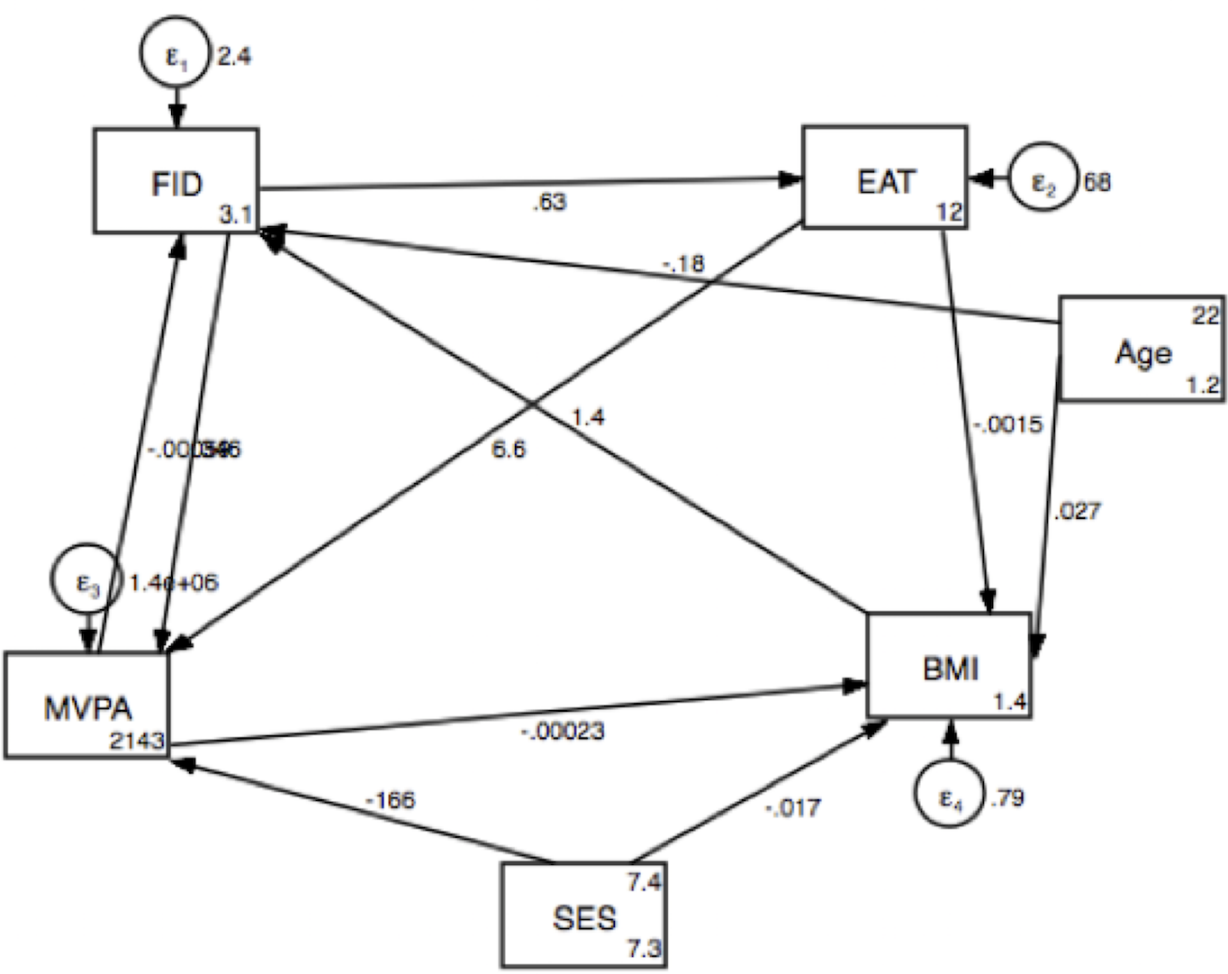
SEM model for the pooled sample.

The pooled SEM model for MVPA, BMI, FID score and EAT score, corrected for SES and age, is presented in Table 5 and Fig 1, and showed a good fit (CFI = 0.945; SRMR = 0.043). FID score was shown to have an overall positive influence on EAT-26 score (p = 0.05), and on MVPA (p = 0.03); although in both cases the indirect effects (via MVPA-BMI and EAT respectively) were significant and negative. Conversely, MVPA negatively influenced FID score (desire to be thinner, p = 0.02)) and EAT-26 score (p = 0.02). FID score directly influenced EAT-26 score (p = 0.01), as well as indirectly and negatively via MVPA (p-0.02) and BMI (p<0.01). BMI had a significant and negative indirect effect on FID score (desire to be thinner) via MVPA (p<0.01); although the overall significant effect of BMI on FID score (desire to be thinner) was positive (p<0.01).

**Table 5:** Results of the SEM model for both sites, pooled.

Exposure: N = 873	Outcome:	Direct effects(95% CI)	Indirect effects (95% CI)	Total effects(95% CI)
**BMI**	FID (desire to be thinner) *Via MVPA*	1.447 (1.029;1.865)*	-0.358 (-0.462;-0.255)*	1.089 (0.774;1.404)*
**MVPA**	FID (desire to be thinner) *Via BMI*	-0.001 (-0.001;-0.00)*	-0.001 (-0.000;0.000)	-0.001 (-0.001;-0.000)*
	BMI *Via FID (desire to be thinner) and EAT (increased risk)*	-0.000 (-0.000;0.000)	0.000 (1.000;0.000)*	-0.000 (-0.000;0.000)
	EAT (increased risk) *Via FID (desire to be thinner)*		-0.000 (-0.000;-0.000)*	-0.000 (-0.000;-0.000)*
**FID** (desire to be thinner)	EAT (increased risk) *Via MVPA and BMI*	0.634 (0.152;1.115)*	-0.156 (-0.295;-0.019)*	0.476 (0.003;0.951)*
	MVPA *Via EAT (increased risk)*	346.4877(37.855;655.101)*	-82.634 (-158.347;-6.921)*	263.843 (30.896;496.791)*
**EAT** (increased risk)	MVPA *Via BMI and FID (desire to be thinner)*	6.570 (-5.627;18.811)	-2.199 (-7.761;3.363)	4.371 (-4.148;12.889)

SRMR = 0.043, CFI = 0.945, chi2 = 0.000, RMSEA = 0.061

* p<0.05

## Discussion

This study examined the differences in body image satisfaction and eating attitudes between rural and urban young adult females in South Africa, and explored the relationships between these disorders, BMI, and physical activity between the two settings and for the group as a whole using site specific regressions and structural equation modeling. We found that overweight and obesity were positively associated with a desire to be thinner and negatively associated with a desire to be fatter in both urban and rural women, and that a body image dissatisfaction predicted higher risk of developing an eating disorder (EAT-26 score >20) in urban females. This tendency towards avoiding overweight was supported by the tendency for urban women to perceive normal weight silhouettes as the “best”, “happiest” and receiving the “most respect” in comparison to an obese silhouette, which they thought received the “least respect”. In contrast, in rural women, risk of developing an eating disorder was associated with a desire to be fatter. As with the urban setting, the majority of rural women viewed a normal weight silhouette as being the “best”; however there was a greater spread in desirability of body size, with more rural than urban women perceiving an overweight or obese silhouette as gaining the “most respect”. This suggests that although westernisation may have influenced body image satisfaction and eating attitudes in young South African women towards a preference for a normal weight silhouette, traditional desirability of higher BMIs in African populations continue to have greater influence over rural compared to urban perceptions.

Increasing prevalence of overweight and obesity in the African context has been associated with rapid urbanisation and a nutrition transition characterised by increased consumption of western diets high in refined carbohydrates, added sugar and fat, as well as reduced physical activity [33, 34]; however we found no difference in mean BMI between the urban and rural settings (25.3 vs. 24.8 kg/m^2^ respectively). Although there were significant differences in the distribution of women across BMI categories, with 7% higher prevalence of overweight and obesity in the urban group, more than a third of rural women were overweight/obese (39%) and there was no difference in underweight prevalence between groups. This suggests that although the effects of lifestyle changes may be more advanced in urban populations, similar patterns of change are occurring in rural settings. This is supported by Mendez et al [35] who showed that, although overweight prevalence was predominantly higher in urban compared to rural adult women from 36 LMICs, rural overweight prevalence was >20% in half of the countries. Although food insecurity may persist in rural South African populations, the increasing accessibility to more affordable energy dense and convenience foods with low micronutrient content may be restructuring the food insecure environment towards a state of “hidden hunger”. Such profiles have been commonly described in urban poor contexts and results from consumption of low quality diets rather than a state of absolute food unavailability and insufficient kilojoule intake [36, 37], and higher consumption of sugar and confectionary has been observed in South African urban compared to rural females [18].

Desirability of a leaner body shape in the urban setting is supported by literature from other parts of Africa—for example, in an older (46(18) years) sample of urban Ghana where overweight and obesity prevalence was high (64.9%), 41.8% preferred a figure smaller than their current size; while being overweight or obese was significantly associated with a desire to lose weight [38]. In Cameroon, although cultural influences persist and the preference towards a larger body size remains—with both men and women perceiving an overweight body size as “normal”—higher BMIs were increasingly associated with a desire to lose weight, particularly in women [5]. Studies from South Africa are largely focused on female adolescents, but are important to consider as adult perceptions and attitudes towards body image and eating may be shaped during this time [39]. A qualitative study by Draper et al [8] found that, although urban South African girls maintain positive perceptions of overweight, there is increasing knowledge and understanding around obesity-associated health risks and the benefits of weight loss. However, cultural and societal stigmatisation of underweight as being a sign of sickness (predominantly HIV and/or TB) remains a strongly influential factor and may create a barrier towards weight loss behaviours. In a sample of South African females with a similar age to the current study sample (mean age = 20), prevalence of body image dissatisfaction was higher in urban compared to rural girls and this was related to risk of developing an eating disorder; but that most overweight or obese females believed that their weight was either normal or overweight [12]. Similar to our findings, Gitau et al [19] showed that at 17 years of age, urban South African females perceive a normal weight silhouette to be the “best”, “happiest” and as “getting respect”, while an underweight silhouette was perceived as being “weak” and an obese silhouette as being the “worst” and “unhappy”. These perceptions were reflected in the emergence of disordered eating attitudes and 16.6% of the adolescent participants were identified as having an EAT-26 score >20 [19]. This is slightly higher than the 12% observed in the urban group in our study; however this difference may be an effect of the younger age of Gitau et al’s sample (mean age 17 vs. 23 years) and of teenage girls being more strongly influenced by external ideals and social norms [40]. Furthermore, the adolescent period is a period of transition often wrought with issues related to body image perception and eating attitudes [22].

Fewer studies have assessed body image satisfaction and eating attitudes in rural contexts; however the present findings around body image perceptions in rural young adult females have been corroborated by other studies in South African adolescent girls who believed a normal weight silhouette to be the “best” and an overweight/obese silhouette to be the “worst” [7]. In the present study, an equal proportion of rural women perceived normal weight and overweight/obese silhouettes as gaining the “most respect”, while the majority of women believed the normal weight silhouette to be “happiest”. The overweight/obese silhouette was perceived as the most “unhappy”; and negative perceptions were associated with underweight and obese body silhouettes equally. As discussed above, these somewhat contradictory perceptions may be due to a strong desire to conform to cultural norms in conjunction with a strong social influence from a transitioning environment during the teenage years [40]. A study by Pedro et al [13] identified the majority of rural South African girls in the sample to be normal weight, and to believe this silhouette to be the “best”, yet 83.5% were still found to exhibit a desire to be thinner (58%) or fatter. Interestingly, those who desired to be fatter had higher BMIs than those who desired to be thinner, and overweight/obese adolescents tended to perceive their own body silhouette to be of a lower BMI than their current size [13]. These findings indicate the growing westernisation and existence of related body image ideals in South Africa, even in rural settings–yet some persistence of preference for a larger body size still exists. Furthermore, black rural young adults from South Africa showed a high prevalence of body image dissatisfaction (yet this was still lower than those observed in urban females in the same study) and were more likely to report their weight to be normal if they were underweight or normal, than if they were overweight or obese [12]. Therefore, although the perception of what constitutes desirable body size seems to be shifting in rural South Africa, overweight/obese girls may have a poor objective perception of their own body size. When coupled with the lingering traditional views of overweight being a sign of prosperity, beauty and good health [41]; the converse perception found in the present study—that obese body silhouettes portray unhappiness and other negative qualities—highlights great conflict between traditional and modern perceptions of health and beauty in this young rural population.

Although the increasing prevalence of disordered eating attitudes in the African setting is commonly attributed to rapid rates of urbanisation and exposure to western ideals, largely through the media, [42, 43] we found a significantly higher proportion of women to be at risk of an eating disorder in the rural compared to the urban setting. In addition, while risk of developing an eating disorder was associated with body image dissatisfaction (particularly a desire to be thinner), in urban females as one would expect, an EAT-26 score ≥20 was only associated with a desire to be fatter in rural females, and body dissatisfaction did not predict EAT-26 score in the regression model as it did for urban females. This, in combination with a Cronbach alpha score indicative of lower internal consistency for EAT-26 in the rural group (Cronbach alpha: 0.64), suggests potential misinterpretation and poor applicability of the questionnaire to rural settings. When examining the questions posed to participants in the EAT-26 questionnaire, we highlighted a number of questions that may be poorly understood in a food insecure environment, particularly considering that only a desire to be fatter was associated with a higher score in rural groups; namely: “I try not to eat when I am hungry”, “I find myself thinking about food a lot”, “I feel that other would prefer it if I ate more” and “I feel that food controls my life”. Although food security was not assessed in this sample, food insecurity persists in South Africa, particularly in rural areas [44]. In settings where a scarcity of food exists at the household level, food preoccupation and restrictive eating behaviours may be common; particularly in females who have documented higher risk of anxiety disorders in response to household food insufficiency than their male counterparts [45]. We therefore propose that, in this case, it is possible that a high EAT-26 score may reflect a need to be cautious about use of food resources in a food insecure environment, rather than a desire to gain or lose weight. This is supported by Pedro et al [13] who similarly showed poor internal consistency of the EAT-26 questionnaire in rural South African adolescents.

The complexity of the interactions between eating attitudes, body image perceptions, BMI, and lifestyle behaviours such as physical activity are difficult to unravel through linear regressions alone, and previous studies mentioned above have tried to partly describe some of these. For example, Mchiza et al [22] attempted to relate body image satisfaction with BMI and self-reported weight control behaviours in South African adolescents and adults in the NHANES national survey, and found that only a small portion of participants attempted to control their weight using exercise or dieting. We thus attempted to elaborate on the interactions between body image satisfaction, physical activity, BMI and eating attitudes through the use of a SEM model. We included SES and age in the model to account for differences between the urban and rural females. The model clearly showed the effect of body image satisfaction on eating attitudes, as well as on physical activity levels. Ultimately, similar to findings from the linear regressions in both groups, and concurrent with the literature, a higher BMI was associated with increased body image dissatisfaction. Body image dissatisfaction (desiring to be thinner) was associated with EAT-26 scores indicative of disordered eating, as well as higher MVPA participation; which was then directly associated with decreased risk of developing a disordered eating attitude and decreased desire to be thinner (i.e.: improved body image satisfaction). This SEM model better explains the interactions between BMI, physical activity levels, eating attitudes and body image satisfaction–physical activity in this sample seems to have beneficial associations with body image satisfaction and eating attitudes, and is potentially used as means to improve body image satisfaction. Meta-analyses have shown that people who exercise more have better body image perceptions, and that increasing physical activity is directly associated with improved body image [46]. On the other end of the spectrum, excessive exercise has been associated with the development and pathogenesis of disordered eating, such as anorexia nervosa [47], and in this light may be a harmful behaviour. In the current study, physical activity was not influenced by eating attitudes, and in fact was associated with a decreased risk of developing an eating disorder. This indicates that, unlike in some cases, physical activity was likely not being abused as part of a disordered mentality around eating, and thus appears to be a healthy behavioural mechanism to control body image satisfaction in this sample. It is important to note that the physical activity variable was constructed of leisure time physical activity, as well as walking for transport, and moderate intensity work. When only considering the leisure time component of physical activity, most participants did not report any participation in leisure time activity, and participation was not associated with body image satisfaction or eating attitudes. It thus appears that physical activity did not need to be voluntary or structured/planned in order to have an effect on body perception. In the NHANES study, nearly 40% of individuals who had attempted to control their weight had done so by increasing physical activity, and most of the rest had adjusted their diet [22]. Since adjusting diet may easily lead to disordered eating behaviours, promotion of healthy levels of physical activity, which does not seem to have a detrimental effect on eating attitudes in this sample of females, may be considered as a tool for improving body image satisfaction in South African females. Interestingly, in this sample, participation in MVPA had no effect on BMI, yet this lack of association with BMI did not alter the direct relationship between body image satisfaction and MVPA. This indicates that weight loss was not a necessary outcome of participation in physical activity for improvement in disordered body image perceptions and attitudes around eating.

While we provide novel data in this study comparing body image satisfaction and eating attitudes in rural and urban young South African women, the poor internal consistency of the EAT-26 questionnaire in the rural group is a limitation that restricts comparability of eating attitudes between settings. In addition, we had no measure of food insecurity or nutritional status for the two groups, which may have provided additional context to the comparisons made between the two populations. Our study was based on self-reported questionnaire data which is prone to bias and may limit the validity of our results, particularly given the nature of body image perceptions and eating attitudes, which are strongly influenced by social pressure and cultural norms [48]. Furthermore, there is potential selection bias as the full BT20+ sample was not included. Lastly, the study sample included predominantly black women and this restricts our ability to generalise findings to other South African ethnicities.

In conclusion our study shows that, although differences exist between body image satisfaction and eating attitudes in urban and rural young female South Africans, with urban women exhibiting a stronger preference towards a lean body shape and a desire to be thinner, westerisation may be starting to affect perceptions and ideals in rural females. As perceptions and attitudes shift, alongside potential declines in the influence of traditional barriers to change, we are provided with an optimal window for interventions that promote a healthy body size. In addition, our findings provide unique evidence to support the potential benefits of physical activity as a means to promote both a healthy body size and image, while reducing the risk of disordered eating. Given the high prevalence of overweight and obesity in both rural and urban women, and increased exposure to modern media, it is critical that women are educated on healthy approaches to weight loss in order to limit emergence of disordered eating attitudes and behaviours.

## Supporting information

S1 Dataset (XLS)

## References

[1] Le Grange D, Telch C, Tibbs J. Eating attitudes and behaviors in 1,435 South African Caucasian and non-Caucasian college students. American Journal of Psychiatry. 1998;155(2):250–4. doi: 10.1176/ajp.155.2.250 9464206 10.1176/ajp.155.2.250

[2] Holmqvist K, Frisén A. Body dissatisfaction across cultures: Findings and research problems. European Eating Disorders Review. 2010;18(2):133–46. doi: 10.1002/erv.965 19806598 10.1002/erv.965

[3] Hidaka BH. Depression as a disease of modernity: explanations for increasing prevalence. Journal of Affective Disorders. 2012;140(3):205–14. doi: 10.1016/j.jad.2011.12.036 22244375 10.1016/j.jad.2011.12.036 PMC3330161

[4] Van Vonderen K, Kinnally W. Media effects on body image: Examining media exposure in the broader context of internal and other social factors. American Communication Journal. 2012;14(2):41–57.

[5] Cohen E, Boetsch G, Palstra F, Pasquet P. Social valorisation of stoutness as a determinant of obesity in the context of nutritional transition in Cameroon: The Bamiléké case. Soc Sci Med. 2013;96:24–32. doi: 10.1016/j.socscimed.2013.07.004 24034948 10.1016/j.socscimed.2013.07.004

[6] Mvo Z, Dick K, Steyn J. Perceptions of overweight African women about acceptable body size of women and children. Curationis 1999:27–31. 10.4102/curationis.v22i2.719 11040616

[7] Gitau T, Micklesfield L, Pettifor J, Norris S. Ethnic Differences in eating attitudes, body image and self esteem among adolescent females living in urban South Africa. J Psychiatry. 2014;17:468–74. doi: 10.4172/2247-2452.1000101

[8] Draper CE, Grobler L, Micklesfield LK, Norris SA. Impact of social norms and social support on diet, physical activity and sedentary behaviour of adolescents: a scoping review. Child Care Health Dev. 2015;41(5):654–67. doi: 10.1111/cch.12241 . 25809525 10.1111/cch.12241

[9] Sharan P, Sundar A. Eating disorders in women. Indian journal of psychiatry. 2015;57(Suppl 2):286–95. 10.4103/0019-5545.161493 PMC4539873 26330646

[10] Szabo C, Allwood C. A cross-cultural study of eating attitudes in adolescent South African females. World Psychiatry. 2004;3(1):41–4. 16633453 PMC1414663

[11] Szabo C, Allwood C. Body figure preference in South African adolescent females: a cross cultural study. African Health Sciences. 2006;6(4):201–6. doi: 10.5555/afhs.2006.6.4.201 17604508 10.5555/afhs.2006.6.4.201 PMC1832064

[12] Senekal M, Steyn N, Mashego T, Nel J. Evaluation of body shape, eating disorders and weight management related parameters in black female students of rural and urban origins. S Afr J Psych. 2001;31(1):45–53.

[13] Pedro T, Micklesfield L, Kahn K, Tollman S, Pettifor J, SA N. Body image satisfaction, eating attitudes and perceptions of female body silhouettes in rural South African adolescents. PLOS ONE. 2016;11(5):e0154784 doi: 10.1371/journal.pone.0154784 27171420 10.1371/journal.pone.0154784 PMC4865095

[14] Shisana O, Labadarios D, Rehle T, Simbayi L, Zuma K, Dhansay A, et al South African National Health and Nutrition Examination Survey (SANHANES-1): 2014 Edition HSRC Press, Cape Town: 2014.

[15] Vorster H. The link between poverty and malnutrition: A South African perspective. Health SA Gesondheid 2010;18(1).

[16] Townsend M, Peerson J, Love B, Achterberg C, Murphy S. Food insecurity is positively related to overweight in women. The Journal of nutrition. 2001;131(6):1738–45. 11385061 10.1093/jn/131.6.1738

[17] Bove C, Olson C. Obesity in low-income rural women: qualitative insights about physical activity and eating patterns. Women & Health. 2006;44(1):57–78. 17182527 10.1300/J013v44n01_04

[18] Steyn N, Senekal M, Brtis S, Nel J. Urban and rural differences in dietary intake, weight status and nutrition knowledge of black female students. Asia Pacific J Cli Nutr. 2000;9:53–9. 10.1046/j.1440-6047.2000.00137.x 24394316

[19] Gitau T, Micklesfield L, Pettifor J, Norris S. Changes in eating attitudes, body esteem and weight control behaviours during adolescence in a South African cohort. PloS One. 2014;9(10):109709 doi: 10.1371/journal.pone.0109709 25310343 10.1371/journal.pone.0109709 PMC4195663

[20] Błachno M, Bryńska A, Tomaszewicz-Libudzic C, Jagielska G, Srebnicki T, Wiśniewski A, et al Obsessive-compulsive symptoms and physical activity in patients with anorexia nervosa–possible relationships. Psychiatr. 2016;50(1):55–64. 10.12740/PP/34810 27086328

[21] Weinstein A, Maayan G, Weinstein Y. A study on the relationship between compulsive exercise, depression and anxiety. Journal of behavioral addictions. 2015;4(4):315–8. doi: 10.1556/2006.4.2015.034 26690627 10.1556/2006.4.2015.034 PMC4712766

[22] Mchiza Z, Parker W, Makoae M, Sewpaul R, Kupamupindi T, Labadarios D. Body Image and weight contorl in South African 15 years and older—SANHANES. BMC Public Health. 2015;15:992 doi: 10.1186/s12889-015-2324-y 26423378 10.1186/s12889-015-2324-y PMC4588465

[23] Kahn K, Collinson MA, Gómez-Olivé FX, Mokoena O, Twine R, Mee P, et al Profile: Agincourt health and socio-demographic surveillance system. Int J Epidemiol. 2012;41(4):988–1001. doi: 10.1093/ije/dys115 22933647 10.1093/ije/dys115 PMC3429877

[24] Richter L, Norris S, Pettifor J, Yach D, Cameron N. Cohort Profile: Mandela's children: the 1990 Birth to Twenty study in South Africa. Int J Epidemiol. 2007;36(3):504–11. doi: 10.1093/ije/dym016 ; PubMed Central PMCID: PMCPMC2702039. 17355979 10.1093/ije/dym016 PMC2702039

[25] Filmer D, Scott K. Assessing Asset Indices. Demography. 2011;49(1):359–92. doi: 10.1007/s13524-011-0077-5 22135117 10.1007/s13524-011-0077-5

[26] Pradeilles R, Griffiths PL, Norris SA, Feeley AB, Rousham EK. Socio-economic influences on anthropometric status in urban South African adolescents: sex differences in the Birth to Twenty Plus cohort. Public Health Nutr. 2015;18(16):2998–3012. doi: 10.1017/S1368980015000415 ; PubMed Central PMCID: PMCPMC4611355. 25757478 10.1017/S1368980015000415 PMC4611355

[27] Mchiza ZJ, Goedecke JH, Lambert EV. Intra-familial and ethnic effects on attitudinal and perceptual body image: a cohort of South African mother-daughter dyads. BMC Public Health. 2011;11:433 doi: 10.1186/1471-2458-11-433 21645339 10.1186/1471-2458-11-433 PMC3138457

[28] Organisation WH. Global Recommendations on Physical Activity for Health. http://www.who.int/dietphysicalactivity/pa/en/index.html2011. 26180873

[29] Vandenberg R. Introduction: Statistical and Methodological Myths and Urban Legends: Where, Pray Tell, Did They Get This Idea? Organisational Research Methods. 2006;9(2):194–201.

[30] Schermelleh-Engel K, Moosbrugger H, Müller H. Evaluating the fit of structural equation models: Tests of significance and descriptive goodness-of-fit measures. Methods of psychological research online. 2003;8(2):23–74.

[31] Hu L, Bentler P. Cutoff criteria for fit indexes in covariance structure analysis: Conventional criteria versus new alternatives. Structural equation modeling: a multidisciplinary journal. 1999;6(1):1–55. doi: 10.1080/10705519909540118

[32] Bhatnagar R, Kim J, Many J. Candidate surveys on program evaluation: Examining Instrument reliability, validity and program effectiveness. American Journal of Educational Research. 2014;2(8):683–90.

[33] Popkin B, Gordon-Larsen P. The nutrition transition: worldwide obesity dynamics and their determinants. Int J Obes. 2004;28(S3):S2–9. 10.1038/sj.ijo.0802804 15543214

[34] Popkin B. Global nutrition dynamics: the world is shifting rapidly toward a diet linked with noncommunicable diseases. Am J Clin Nutr. 2006;84(2):289–98. 16895874 10.1093/ajcn/84.1.289

[35] Mendez M, Monteiro C, Popkin B. Overweight exceeds underweight among women in most developing countries. Am J Clin Nutr. 2005;81(3):714–21. 15755843 10.1093/ajcn/81.3.714

[36] Wurwarg J. Urbanization and hunger: food policies and programs, responding to urbanization, and benefiting the urban poor in three cities. J Int Aff N Y. 2014;67(2):75–107.

[37] Roetter R, Keulen H. Food Security In: Science for Agriculture and Rural Development in Low-income Countries. Roetter RP KH, Kuiper M, Verhagen J, Laar HHV, editor. Springer, Netherlands 2007.

[38] Benkeser R, Biritwum R, Hill A. Prevalence of overweight and obesity and perception of healthy and desirable body size in urban, Ghanaian women. Ghana Med J. 2012;46(2):66–75. 22942454 PMC3426384

[39] Bucchianeri M, Arikian A, Hannan P, Eisenberg M, Neumark-Sztainer D. Body dissatisfaction from adolescence to young adulthood: findings from a 10-year longitudinal study. Body Image. 2013;10(1): doi: 10.1016/j.bodyim.2012.09.001 23084464 10.1016/j.bodyim.2012.09.001 PMC3814026

[40] Knoll L, Magis-Weinberg L, Speekenbrink M, Blakemore S. Social influence on risk perception during adolescence. Psychol Sci. 2015;26(5):583–92. doi: 10.1177/0956797615569578 25810453 10.1177/0956797615569578 PMC4426139

[41] Puoane T, Fourie J, Shapiro M, Rosling L, Tshaka N, Oelefse A. “Big is beautiful”–an exploration with urban black community health workers in a South African township. South Afr J Clin Nutr. 2015;18(1):6–15.

[42] Son G, Hoeken D, Bartelds A, Furth E, Hoek H. Urbanisation and the incidence of eating disorders. Br J Psychiatry. 2006;89(6):562–3. 10.1192/bjp.bp.106.021378 17139044

[43] Chisuwa N, O’Dea J. Body image and eating disorders amongst Japanese adolescents. A review of the literature. Appetite. 2010;54(1):5–15. doi: 10.1016/j.appet.2009.11.008 19941921 10.1016/j.appet.2009.11.008

[44] Labadarios D, Mchiza ZJ-R, Steyn NP, Gericke G, Maunder EMW, Davids YD, et al Food security in South Africa: a review of national surveys. Bulletin of the World Health Organization. 2011;89:891–9. doi: 10.2471/BLT.11.089243 22271946 10.2471/BLT.11.089243 PMC3260897

[45] Sorsdahl K, Slopen N, Siefert K, Seedat S, Stein D, Williams D. Household food insufficiency and mental health in South Africa. J Epidemiol Community Health. 2011;65(5):426–31. doi: 10.1136/jech.2009.091462 20427548 10.1136/jech.2009.091462 PMC3195371

[46] Hausenblas H, Fallon E. Exercise and body image: A meta-analysis. Psychology & Health. 2006;21(1):33–47. doi: 10.1080/14768320500105270

[47] Davis C, Kennedy SH, Ravelski E, M. D. The role of physical activity in the development and maintenance of eating disorders. Psychol Med. 1994;24(4):957–67. 7892363 10.1017/s0033291700029044

[48] Van de Mortel T. Faking it: social desirability response bias in self-report research. Aust J Adv Nurs. 2008;25(4):40.

